# Referral Time of Advance Cancer Patients to Palliative Care Services and Its Predictors in Specialized Cancer Center

**DOI:** 10.7759/cureus.12300

**Published:** 2020-12-26

**Authors:** Nabil ALMouaalamy, Khaled AlMarwani, Abdulmajeed AlMehmadi, Ahmed A AlNakhli, Yasser AlGhamdi, Abdullah Zarkan, Alaa Althubaiti

**Affiliations:** 1 Princess Noorah Oncology Center/Palliative Care Service, King Abdulaziz Medical City, National Guard Health Affairs, Jeddah, SAU; 2 Research, King Abdullah International Medical Research Center, Jeddah, SAU; 3 College of Medicine, King Saud Bin Abdulaziz University for Health Sciences, Jeddah, SAU

**Keywords:** palliative care services, length of stay, referral time, cancer

## Abstract

Background and aim

Globally, there is a discrepancy in whether terminal cancer patients are early referred to palliative care service (PCS) or not. A late referral can lead to a delay in treating and palliating those patients in need. The aim of this study is to investigate the referral time patterns of advanced cancer patients to PCS in Princess Noorah Oncology Center (PNOC) at King Abdulaziz Medical City, Ministry of National Guard Health Affairs, Jeddah, Saudi Arabia. In addition, this study evaluates the factors that influence referral time to the palliative care unit (PCU), along with the overall survival rate.

Methods

This was a retrospective cross-sectional study (chart review) conducted at the inpatient unit in PNOC and included all patients referred to PCS between January 1st, 2016, and December 31, 2016. In total, 153 patients met the inclusion criteria, and their data were collected and analyzed.

Results

The median length of stay (LOS) was five days (95% CI: 3.85-6.15). Among the 153 patients, 22 (14.4%) died within 24 hours of enrollment to PCU. Patients who were referred to the PCU with non-metastasis disease had a median LOS of nine days, which is significantly longer than the median LOS in patients with metastatic disease (95% CI: 0.35-0.82, p=0.004), which indicates that they referred relatively earlier to PCU. The hazard ratio for death in patients with non-metastatic cancer stage was 0.54 (95% CI: 0.35-0.82, p=0.004).

Conclusion

Referral of advanced cancer patients to palliative care services occurs late in their disease course in our institution, like other institutions, with variation in LOS, which shorten their stay at palliative, as well as, affects their quality of life (QOL) and ability to plan or make a decision regarding their care. Education of the public and, most importantly, the medical community is needed.

## Introduction

Palliative care services (PCS) was first established in Saudi Arabia in 1989 at King Faisal Specialist Hospital and Research Centre in Riyadh [1]. Since then, it has been expanding slowly to other parts of the country. Nonetheless, palliative care services in Saudi Arabia is still, comparatively, in their early stages. As a field, palliative care is defined by the World Health Organization (WHO) as an approach that concerns itself with the improvement of quality of life (QOL) of patients with life-threatening diseases. Palliative care aims to ameliorate patients’ physical symptoms, such as pain, and relieve suffering. However, treating physical symptoms is not the only element of palliative care. Palliative care also provides spiritual as well as psychosocial support by encouraging patients to live actively instead of passively waiting for death [1, 2]. Many studies revealed a wide range of advantages of early palliative care, such as improving patients' QOL, decreasing end-of-life aggressive care, and reducing medical costs [3]. Furthermore, early referral to PCS has been associated with fewer emergency department visits, hospitalizations, and deaths in the hospital during the last 30 days of life [4]. In contrast, a late referral was found to be a sign of the poor quality of care [3-5].

Globally, there is a discrepancy in whether terminal cancer patients are early referred to PCS or not. That is reflected by the length of stay (LOS), which is the duration of time from the patient’s referral to PCS until the patient’s death. The longer LOS indicates early referral to PCS, and the shorter length of stay indicates late referral. The length of stay was variant among different countries. For instance, Australia has 54 days of LOS [6]. While in the United States, it was 26 days [7]. In Italy and Korea, it was 37.9 days and 18 days, respectively [7, 8]. The Chinese study found out that the advanced cancer patients who were admitted to the palliative care unit (PCU) had a median of 21 days length of stay (LOS) in the unit before death, which was considered as a late referral according to their definitions and compared to other international PCUs [9].

Locally, referral time has not been investigated intensely. Alshammary et al. study conducted in King Fahad Medical City’s palliative care department, Riyadh, Saudi Arabia, reported that the median LOS was 19 days for non-hematological cancer patients [10]. Alsirafy et al. study carried out in King Faisal Specialist Hospital and Research Center in Riyadh, Saudi Arabia, found that the median LOS was also 19 days, which is considered relatively shorter compared to the developed countries [11].

To the limits of our knowledge, no study has investigated the LOS in palliative care in the western region of Saudi Arabia. Therefore, this study aims to investigate the referral time patterns of advanced cancer patients to PCS in Princess Noura Oncology Center (PNOC) at King Abdulaziz Medical City, Ministry of National Guard Health Affairs, Jeddah, Saudi Arabia. Also, this study evaluates the factors that influence referral time to PCU, along with the overall survival rate. Our study is significant in improving hospital policies to encourage early referral to the palliative care department, hence, improving patients care.

## Materials and methods

This was a retrospective cross-sectional study conducted at PNOC at King Abdulaziz Medical City, Ministry of National Guard Health Affairs, Jeddah City. PNOC is a tertiary cancer center with an 88-bed Adult Oncology Inpatient Department, 22-bed Bone Marrow Transplant Unit, 32-bed Pediatric Hematology-Oncology services including Pediatric Bone Marrow Transplant (BMT), and specialized Pediatric Oncology Emergency Room (POWER). The palliative care service is integrated within the PNOC.

The database was searched to identify all cancer patients aged 14 years or older who were referred to the PCU in 2016. We included both female and male patients. All patients who were alive at the time of the data collection or patients with missing data were excluded. The data was collected using a data collection sheet in which the following variables were included: 1) demographic characteristics including age, sex, marital status, and area of residence; 2) medical problems of the patient including, primary cancer type, metastasis, admitting diagnosis, the setting of referral, type of received treatment and purpose of referral to PCU; 3) length of stay in PCU - the shorter LOS duration indicates late referral time to PCU and the longer LOS duration indicates early referral time to PCU; 4) overall survival from patient’s enrollment to PCU to patient’s death. The data was collected from the electronic health records (BestCare System) and paper-based records.

Frequencies and percentages were used to describe categorical variables, while mean, standard deviation, median, and range (min to max) were used for continuous variables. Survival was calculated from the time of admission to PCS to the time of death and estimated using the Kaplan-Meier method. Additional univariate analysis was performed to determine the predictors of time to death using the Cox proportional hazards regression model. All p-values in the univariate analysis were above 0.2 except for one factor; therefore, no multivariate Cox proportional hazards regression model was developed. The Chi-squared test and Fisher exact test were used for the bivariate analysis to explore the association between demographic and clinical characteristics and the patient’s number of admissions. A p-value <0.05 was considered to be statistically significant. All statistical analyses were performed using IBM SPSS Statistics software, version 23.0 (IBM Inc., Armonk, USA).

## Results

Table 1 shows patient characteristics. In total, 153 patients met the inclusion criteria and were referred to the PCU. The mean age was 60.51 ± 16.7 years; 77 (50.3%) were female. Among the patients, 120 (78.4%) patients were admitted once, and 33 (21.6%) admitted more than once. The most common primary cancer types were breast cancer 26 (17%), colorectal cancer 15 (9.8%), and pancreatic cancer 10 (6.5%). Approximately two-thirds of the patients (n=108, 70.6%) had metastatic at the time of referral to PCU, and 135 (88.2%) had a non-hematological malignancy. The majority of the patients (n=148, 96.7%) were on the do-not-resuscitate (DNR) order. Fifty-three patients (34.6%) received no treatment, 41 (26.8%) received only chemotherapy, and 22 (14.4%) had a combination of surgery and chemotherapy. Among the referring specialties in regards to having the most point of referral to PCU, medical oncology takes the lead with 59 (38.6%) referrals, followed by emergency medicine 31 (20.3%), and general medicine 21 (13.7%). The median number of admissions is one (range 1-4).

**Table 1:** General characteristics of patients HTN - hypertension; DM - diabetes mellitus; CVA - cerebrovascular accident; HF - heart failure; PCU - palliative care unit; CKD - chronic kidney disease; DNR - do not resuscitate

Characteristic	n	%	Characteristic	n	%
Age			Received treatment		
≤61	77	50.3	Chemotherapy	41	26.8
>61	76	49.7	Surgical	10	6.5
Sex			Radiotherapy	5	3.3
Male	76	49.7	Chemotherapy and surgery	22	14.4
Female	77	50.3	Chemotherapy and radiotherapy	10	6.5
Type of cancer			Radiotherapy and surgery	4	2.6
Breast cancer	26	17	All of them	8	5.2
Colorectal	15	9.8	No treatment	53	34.6
Cervical	5	3.3	Form of cancer		
Lung	8	5.2	Hematological	18	11.8
Stomach / liver	7 / 9	4.6 / 4.9	Non-hematological stage of cancer	135	88.2
Pancreas	10	6.5	Metastatic	108	70.6
Esophageal	3	2	Non-metastatic	34	22.2
Urological	9	5.9	Comorbidities		
Sarcoma	3	2	HTN		
Ovarian	5	3.3	Yes	60	39.2
Head and neck	9	5.9	No	93	6.8
Gallbladder	4	2.6	DM		
Lymphomas	9	5.9	Yes	53	34.6
Other	31	20.3	No	100	65.4
Patients with CVA			CKD		
Yes	6	3.9	Yes	12	6.8
No	147	96.1	No	141	92.2
Patients with HF			Code status		
Yes	5	3.3	DNR	148	96.7
No	148	96.7	Full code	5	3.3
Number of PCU admissions					
One admission	120	78.4	Three admissions	7	4.6
Two admissions	23	15	Four admissions	3	2

Survival

**Figure 1:**
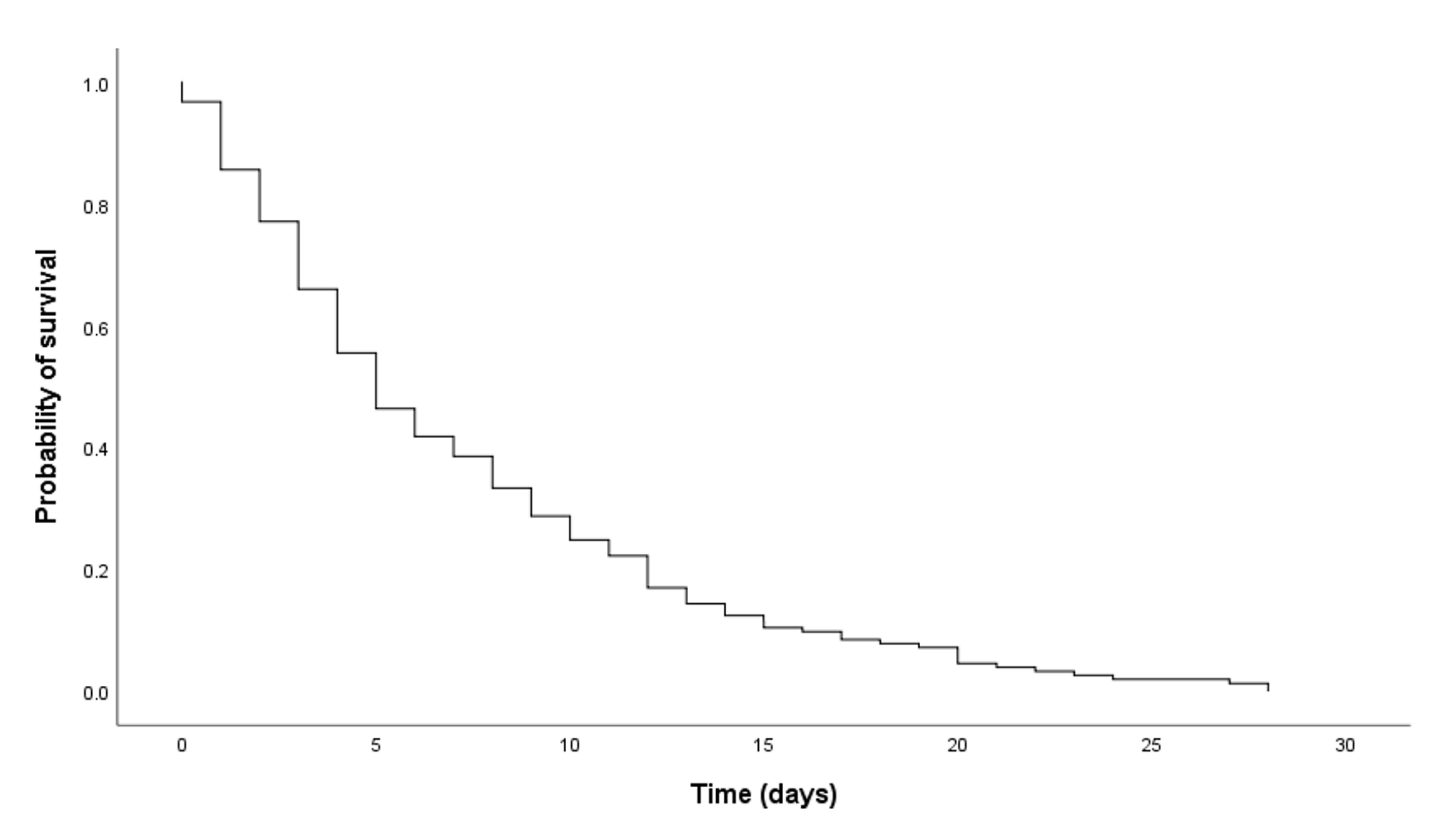
Kaplan-Meier curve (N=153)

Figure 1 illustrates the Kaplan-Meier curve of survival measured from the day of first PCU admission to the day of death (in days). The median LOS was five days (95% CI: 3.85-6.15). Among the 153 patients, 22 (14.4%) died within 24 hours of enrollment to PCU.

Association of clinical and demographic characteristics with LOS

Patients who were referred to the PCU with non-metastasis disease had a median LOS of nine days, which is significantly longer than the median LOS in patients with metastatic disease (95% CI: 0.35-0.82, p=0.004), which indicates that they referred relatively earlier to PCU. The hazard ratio for death in patients with non-metastatic cancer stage was 0.54 (95% CI: 0.35-0.82, p=0.004). It means that patients with non-metastatic cancer at any time point during the studied period of time were about 50% less likely to die than patients with metastatic cancer upon admission to PCU. Other variables, such as age, sex, and cancer type, did not affect the LOS (Table 2). Since only the cancer stage affected LOS, and all p-values for other variables were above 0.2, no multivariate analysis was conducted.

**Table 2:** Cox proportional hazards regression analysis PCS - palliative care service

Variable	LOS (days); median (range)	HR	95% CI	P-value
Age		0.84	0.61-1.16	0.286
≤61	4 (0-28)			
>61	5.5 (0-28)			
Gender		0.77	0.95-1.8	0.204
Male	6 (1-28)			
Female	5(0-28)			
Stage of cancer		0.54	0.35-0.82	0.004
Metastatic	5 (0-21)			
Non-metastatic	9 (0-28)			
Comorbidities		0.89	0.64-1.22	0.467
None	5 (0-28)			
Yes	5 (0-28)			
Cancer form type		0.89	0.54-1.50	0.644
Hematological	3.5 (1-23)			
Non-hematological	5 (0-28)			
Number of admissions		0.98	0.66-1.44	0.897
Admitted once	5 (0-28)			
More than one	7 (0-23)			
Treatment		1.15	0.82-1.61	0.415
No	7 (0-28)			
Yes	5 (0-28)			
Purpose of referral to PCS Admission		1.1	0.70-1.56	0.826
Under PCS physician	5 (0-28)			
Other	5 (0-28)			

Association between the number of admission and patients’ characteristics

The associations between the number of admissions and different variables are shown in Table 3. The number of PCU admissions was significantly associated with the purpose of referral. Patients referred for admission under PCU physician were more likely to be admitted more than once compared to those referred to PCU for other reasons (51.6% vs. 13.9%, p<0.001). Likewise, patients aged less than 61 were more likely to be admitted more than once compared to those aged more than 61 (29.9% vs. 13.2%, p=0.012). Additionally, patients who received no treatment for their cancer were more likely to be admitted more than once (7.4% vs. 29%, p=0.002).

**Table 3:** Comparison of patients’ characteristics between number of admissions (N=153) PCS - palliative care services; PCU - palliative care unit

Variable	Admitted once (n=120)	Admitted more than once (n=33)	P-value
Age, years			
≤61	54 (70.1)	23 (29.9)	0.012
>61	66 (86.8)	10 (13.2)	
Sex			
Male	60 (78.9)	16 (21.1)	0.877
Female	60 (77.9)	17 (22.1)	
Stage of cancer			
Metastatic	84 (77.8)	24 (22.2)	0.874
Non-metastatic	26 (76.5)	8 (23.5)	
Comorbidity			
No	57 (71.3)	23 (28.7)	0.024
Yes	63 (86.3)	10 (13.7)	
Cancer form type			
Hematological	14 (77.8)	4 (22.2)	0.943
Non-hematological	106 (78.5)	29 (21.5)	
Treatment			
No	49 (92.5)	4 (7.5)	0.002
Yes	71 (71)	29 (29)	
Purpose of referral to PCS			
Admission under PCS physician	15 (48.4)	16 (51.6)	<0.001
Other	105 (86.1)	17 (13.9)	

Purposes of referral to PCU 

In PNOC, there are four purposes for referral a cancer patient to the PNOC palliative care unit. First, consultation and concurrent care, which means that the palliative care physician is consulted at the same time the patient receives care under oncologist - concurrent oncology palliative care - and this purpose of referral constitutes more than half of our sample (n=85, 55.6%). Second, consultation and transfer of care, which means that the palliative care physician is consulted but for the purpose of transferring the patient to PCS - this group makes up 35 patients (22.9%). Third, patients who were admitted under the palliative care physicians, which constitute 31 patients (22.3%). The last purpose of referral a cancer patient to palliative care services is a consultation only - this constitutes only two patients (1.3%). Table 4 shows the purposes of referral to PCU.

**Table 4:** Purposes of referral to PCU PCU - palliative care unit

Purpose	Descriptive statistics n (%)
Admission under palliative care physician	31 (20.3)
Consultation and transfer of care	35 (22.9)
Consultation and concurrent care	85 (55.6)
Consultation only	2 (1.3)

## Discussion

To our knowledge, this study is the first to examine the length of stay of cancer patients in palliative care and its predictors in the western region of Saudi Arabia. This study showed that the referral of cancer patients to the palliative care unit is very late. Locally, two studies conducted in Riyadh reported that the LOS was 19 days [10, 11]. In our study, the median LOS was only five days, which is the shortest among these local studies as well as global studies that were conducted in the United States (42 days), Australia (54 days), and China (21 days) [6-9]. Late referral may prevent patients from benefiting from the integral part of palliative care services, which is to alleviate patients' suffering and help them and their caregivers experience the highest quality of life [2].

In this study, the vast majority of patients (70.6%) who were referred to palliative care had metastasis, which was a significant factor that affected the length of stay. This finding is consistent with two studies in terms of the majority of the patients who referred to PCU showed up with metastasis (95% and 84.3%, respectively) [9, 10]. A study was conducted to predict the survival of 827 newly referred patients based on 17 demographic and clinical factors. The study revealed that metastasis was the leading factor that accurately predicted survival with a mean score of more than eight [12-15].

Many factors may contribute to the fact that we ended up having a late referral to palliative care. Such factors can be mainly attributed to the health-care system as a whole, where there is an obvious lack of hospice and palliative medicine understanding [13]. A study was done among college students to measure the awareness of hospice care and services in Saudi Arabia. The study revealed that 74 (86.05%) of 86 Saudi Arabian students attending a Mid-Western public university did not know what hospice care is, and only 12 (13.95%) of 86 Saudi Arabian students had some knowledge about hospice care [16].

The public’s misperceptions of palliative care underline some of the key factors influencing the early implementation of palliative care. In one study, a large percentage of people (73.9%) associated palliative care with end of life care, while others considered this type of service as only for the elderly; some participants even considered it as euthanasia [17]. Another report has shown that as many as 42.7% of participants, who self-report as "know a lot" about palliative care, agree with the statement "when I think of palliative care, I automatically think of death" [18].

For many people, palliative care and hospice care are synonymous. The misperception is possibly due to the many similarities between them, including management of symptoms, interdisciplinary care, and caregiver involvement. However, some differences distinguish hospice care from any other care; these include the involvement of hospice care after cessation of treatment, while in the case of palliative care, it is implemented concurrently with treatment. Furthermore, hospice care is applied when the expected survival is less than six months. Also, hospice care provides bereavement support to the families to help them cope with losing their loved one [16-18].

Length of stay

The LOS spectrum can range between having a (long-short) period at the PCU depending on the factor. For instance, we found that the hematological factor has the shortest median LOS of 3.5 days (95% CI: 0.54-1.50, p=0.644), which means, if any patient - at any time point during the studied period - was admitted to PCU with a hematological tumor form factor, the patient is more likely to have a probability of survival by 11%. In contrast, patients who were admitted to PCU more than once and/or had no treatment have a longer median LOS of seven days than the hematological group, but shorter median LOS compared to the non-metastatic group. However, the only significant factor we have in this study, as stated before, is the stage of cancer at the time of referral to PCU (metastatic and non-metastatic), where the metastatic median LOS is five days, and the non-metastatic median LOS is nine days. Furthermore, if a patient was admitted to PCU - at any time point during the studied period - with non-metastatic staging, he/she will have a probability of survival by 46% compared to all other groups that showed lower survival rates. The American Society of Clinical Oncology concluded in their recommendation that "metastatic cancer or high symptom burden" patients should be integrated early in palliative care [19]. Having said that, on a survey that had been conducted on European medical oncologists attitudes towards palliative care for advanced and incurable cancer patients, they found that 75% of oncologists either agreed or strongly agreed that advanced cancer patients should "receive concurrent palliative care"; however, a significant consensus of up to 75% of the medical oncologists agreed that "the best person to coordinate the palliative care to an advanced cancer patient is the medical oncologist" [20, 21]. Other reports have shown poor communication between oncologists and palliative care providers, ultimately reducing PCS utilization [22].

Referring specialty

The setting of point of care referral appeared to be a crucial indicator of end-of-life quality (EOL) for patients. This study found that medical oncology referral was the first point of referral to PCU (38.6%), followed by emergency medicine department (ED; 20.3%) and general medicine (13.7%). While the inpatient referral has been proven to be an effective measure to reduce the burden of suffering by many studies, a referral from ED is considered a poor indicator of QOL mainly because patients are aggressively treated due to lack of time [4]. Hematology and gynecologic oncology ranked fourth in our study, with 7.2% of referrals, followed by surgery with 5.2%, and radiation oncology with 3.9% of referrals.

One study that surveyed the attitude of (hematologic vs. solid tumor medical) oncologists towards EOL and patient access to palliative care found that only 14% of oncologists are comfortable referring advanced cancer patients, who receive active treatment, to palliative care; while this percentage increase to 24% if the patients are no longer receiving cancer treatment. The study concluded that hematologic oncologists expressed a "lower comfortable level" referring patients to EOL care compared to solid tumor oncologists, which might contribute to having lower access to EOL care [21, 22].

Integration of palliative care into primary care can significantly impact early referral to palliative care by wisely addressing the need for such care. Many general physicians have worked with patients and their caregivers for a reasonable amount of time and have the additional advantage of well-established relationships. Therefore, those general physicians can identify patients who may benefit from any palliative care service [14].

In Saudi Arabia, there are also unique challenges that could prevent the early involvement of palliative care, such as scarcity of palliative care centers and the restrictive policies regarding opioid availability [23]. Moreover, the field of palliative care is relatively new in Saudi Arabia; thus, it is not widely acknowledged by the public and the medical community alike. Also, many people live in rural areas who lack awareness of medical services [10]. Therefore, increasing the number of palliative care centers would facilitate easy access to PCS.

Our PCU setting

The PCS at PNOC was founded formally in 2001 with the initiation of the Oncology Center. It serves both adult and pediatric patients who need palliative care. In our setting at PNOC, patients are admitted to PCU either directly under a palliative care physician's approval or through one of the three types of consultation purpose referrals in the hospital. These consultation referrals are as follows.

Consultation and transfer of care where the patient has to completely fulfill the PCU criteria to be admitted. The second purpose of the referral is called consultation and concurrent care, where the patient is followed up regularly regarding special issues. The third purpose is when the PCU physician is asked for consultations only. In this study, patients admitted via a palliative care physician were markedly associated with more than one admission to PCU compared to those who were referred (51.6% vs. 13.9%, p<0.001), which make sense as the palliative care patients are in more advance stage of the disease and tend to be sicker in general but also lacking hospice care and appropriate home care support can be a factor as well [16].

In one qualitative interview study that supplemented the ENABLE (Educate, Nurture, Advise, Before Life Ends) II randomized controlled trial (RCT), describing the oncologists’ experiences in caring for advanced cancer patients in a "well-integrated concurrent oncology palliative care environment", most oncologists considered a palliative care referral of a newly-diagnosed advanced cancer patient. However, on the PCS consultation or co-management phase, PCS will be limited to either consultation or co-managing of the patient’s care, such as "difficult physical problems, pain management, or, nausea". Despite that, oncologists made it clear that they did not wish to "give up" the patient's care. In the next phase, where palliative care shares the load, a PCS will rely on patients with "complex care needs" [24].

Overcoming these obstacles requires a constructive and well-planned team approach in order to keep the patient as comfortable as possible until the day of death. Relieving pain and symptoms only should not be the ultimate goal for a patient who is facing an incurable illness; however, an extensive management plan and support system should be implemented in our region in order to improve the QOL for all patients [2].

Treatment options

In terms of the previous treatment received by the patient, the treatment regimen ranged between no treatment, chemotherapy, radiotherapy, surgery, or a combination, where 53 patients (34.6%) received no treatment, which means they got only palliative care, symptoms relief, pain reduce and improve the QOL because they are not fit for therapy for various reasons; 41 (26.8%) received chemotherapy alone, 22 (14.4%) had a combination of surgery and chemotherapy, 10 (6.5%) had either surgery or a combination of chemotherapy and radiotherapy. While eight patients (5.2%) received all therapies (chemotherapy, radiotherapy, and surgery), whilst five patients (3.3%) had radiotherapy alone, and four patients (2.6%) combined radiotherapy and surgery. Data on neoadjuvant or adjuvant chemotherapy courses or the chronological order of the given therapies were not collected due to missing data in some files. In one retrospective study that recruited 759 patients, chemotherapy was used in 56% of the cases with a median of three-courses, 52.6% had surgery, 30% had radiotherapy, and 18.7% received other anticancer therapy as radiofrequency ablation, peritoneal perfusion, and clinical trials [9]. Future studies should address whether or not a multi or solo aggressive cancer treatment has a role or relation to the length of stay of a patient in the palliative care unit.

Limitations

This research, however, is subjected to several limitations that shall be noted. First, since it is hard to follow-up cancer patients outside the institution, we excluded all cancer patients who were admitted but died outside the palliative care unit, which affected our sample size. Second, although our results are consistent with others’ locally conducted studies [10-11] in terms of shorter LOS before death, it is based on a single institution and cannot be generalized all over the PCUs in the region. Third, a big part of our data was paper-based and had some critical missing information for marking the LOS, especially the comorbidities and cancer diagnosis date. Addressing a detailed list of the comorbidities and the patient’s date of cancer diagnosis may help determine the relative LOS at the PCU in future studies.

## Conclusions

Referral of advanced cancer patients to palliative care services occurs late in their disease course in PNOC. Like in other institutions, variation in LOS shortens patients' stay at PNOC PCU as well as affects their QOL and ability to plan or make a decision regarding their care. Education of the public and, most importantly, the medical community is needed in regard to integration and early utilization of palliative care services in advanced cancer patient’s medical plans. Further research is required to investigate other factors that affect late referral to palliative care such as the attitude of doctors toward palliative care and the ideal timing of advanced cancer patient referral.
